# Supplementary material for: Molecular de-novo design through deep reinforcement learning
Source: J Cheminform. 2017 Sep 4;9:48. doi: 10.1186/s13321-017-0235-x (PMC5583141; doi:10.1186/s13321-017-0235-x)
Supplement: Supplementary file 1 — Additional file 1. Equivalence to REINFORCE. Proof that the method used can be described as a REINFORCE type algorithm. [file 13321_2017_235_MOESM1_ESM.pdf]

1 Additional File 1.

Here is shown how the cost function of the form $J(\theta) = [\log P(A)_{\mathbb{A}} - \log P(A)_{\mathbb{U}}]^2$ can be expressed as a REINFORCE algorithm with a single point estimate of the return $G(a_t, s_t) = [\log P(A)_{\mathbb{A}} - \log P(A)_{\mathbb{U}}]^2 / \log P(A)_{\mathbb{A}}$. A REINFORCE type algorithm for an episode $\{s_0, a_0, r_0, ..., s_T, a_T, r_T\}$ following the stochastic policy π_θ is described by the update rule:

$$\theta = \theta - \alpha \nabla_\theta \sum_{t=0}^{T} \log \pi_\theta(a_t \mid s_t)(G(a_t, s_t) - b)$$

Where α in this case is the step size, b is the reward baseline, and $G(a_t, s_t) = \sum_t^T (r_t)$ is the observed cumulative reward from time t until the end of the episode. In a REINFORCE algorithm this single sampled trajectory serves as an unbiased estimator of the expected cumulative reward. If we use a zero baseline $b = 0$, this is equivalent to the cost function:

$$J(\theta) = \sum_{t=0}^{T} \log \pi_\theta(a_t \mid s_t) G(a_t, s_t)$$

If we define the reward for any state-action pair during the episode as equal to 0 except for the last step where it is $G(A)$, this expression can be written as:

$$\forall t \in [0, ..., T], G(a_t, s_t) = \sum_t^T (r_t) = G(A)$$

The cost function becomes:

$$J(\theta) = \sum_{t=0}^{T} \log \pi_\theta(a_t \mid s_t) G(a_t, s_t) = G(A) \sum_{t=0}^{T} \log \pi_\theta(a_t \mid s_t)$$

We now note that the sum $\sum_{t=0}^{T} \log \pi_\theta(a_t \mid s_t)$ is equal to the Agent likelihood for the sequence:

$$J(\theta) = G(A) \sum_{t=0}^{T} \log \pi_\theta(a_t \mid s_t) = G(A) \log P(A)_{\mathbb{A}}$$

If we choose the reward for the final step of the sequence A to be $G(A) = [\log P(A)_{\mathbb{A}} - \log P(A)_{\mathbb{U}}]^2 / \log P(A)_{\mathbb{A}}$, we recover our initial cost function:

$$J(\theta) = \frac{[\log P(A)_{\mathbb{A}} - \log P(A)_{\mathbb{U}}]^2}{\log P(A)_{\mathbb{A}}} \log P(A)_{\mathbb{A}} = [\log P(A)_{\mathbb{A}} - \log P(A)_{\mathbb{U}}]^2$$

■
