# Supplementary material for: Molecular de-novo design through deep reinforcement learning
Source: J Cheminform. 2017 Sep 4;9:48. doi: 10.1186/s13321-017-0235-x (PMC5583141; doi:10.1186/s13321-017-0235-x)
Supplement: Supplementary file 2 — Additional file 2. Generated structures. Structures generated by the canonical Prior and different Agents. [file 13321_2017_235_MOESM2_ESM.pdf]

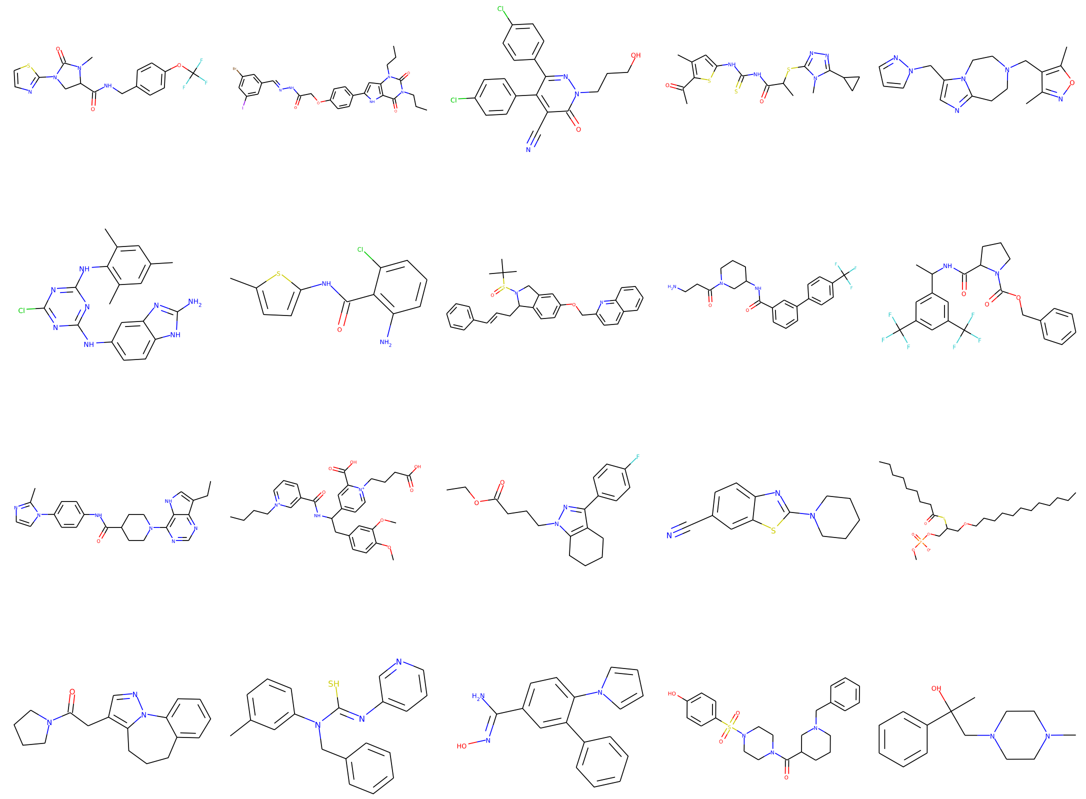

Figure 10 Additional file 2.1 Randomly selected structures generated by the canonical Prior.

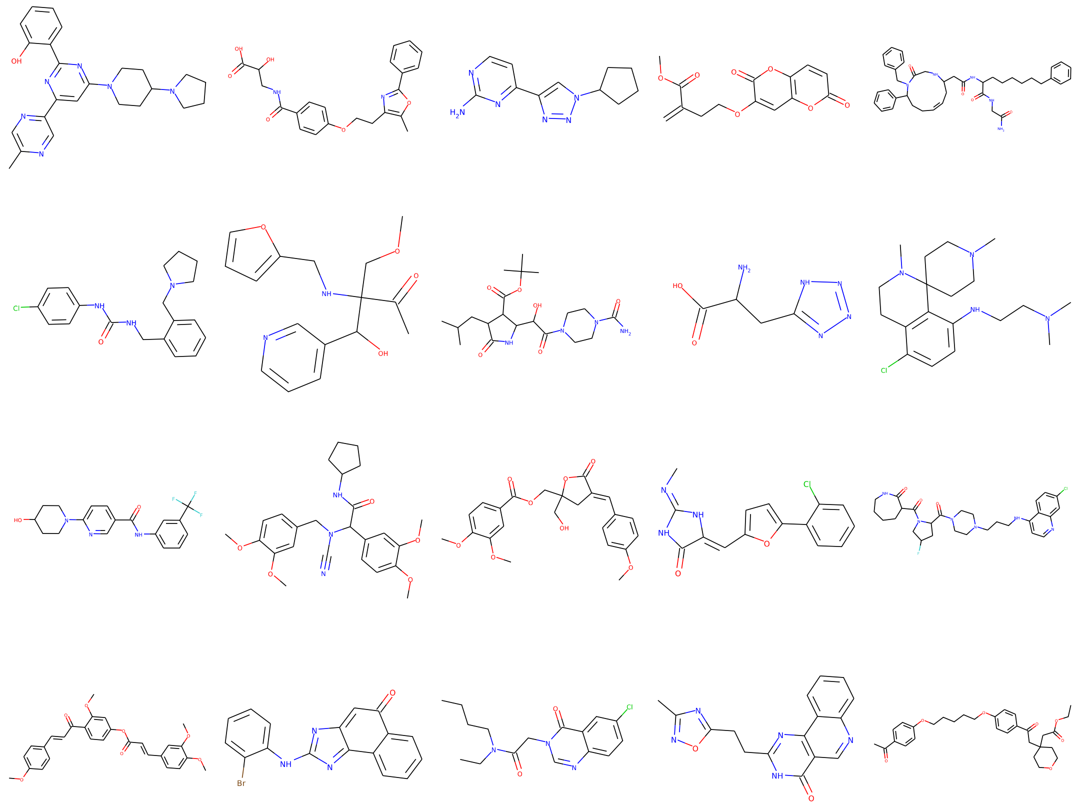

Figure 11 Additional file 2.2 Randomly selected structures generated by the Agent trained to avoid sulphur.

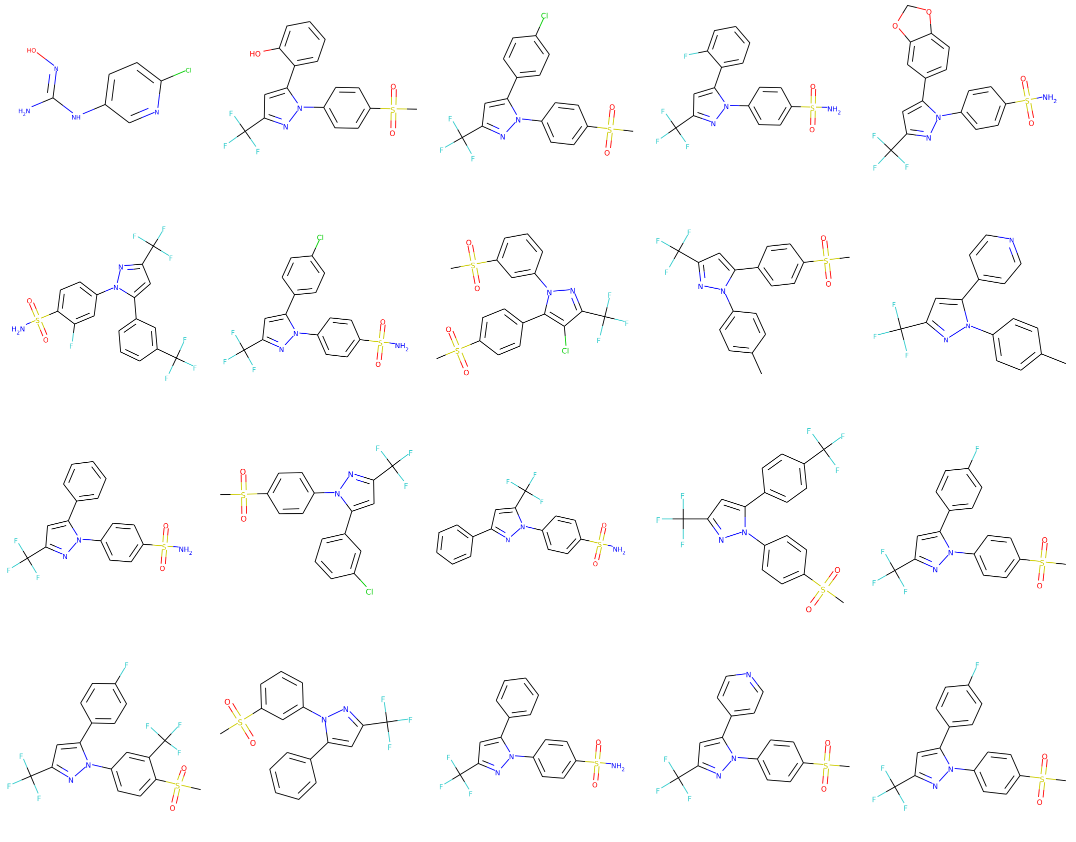

Figure 12 Additional file 2.3 Randomly selected structures generated by the Agent based on the reduced Prior trained to design analogues of Celecoxib.

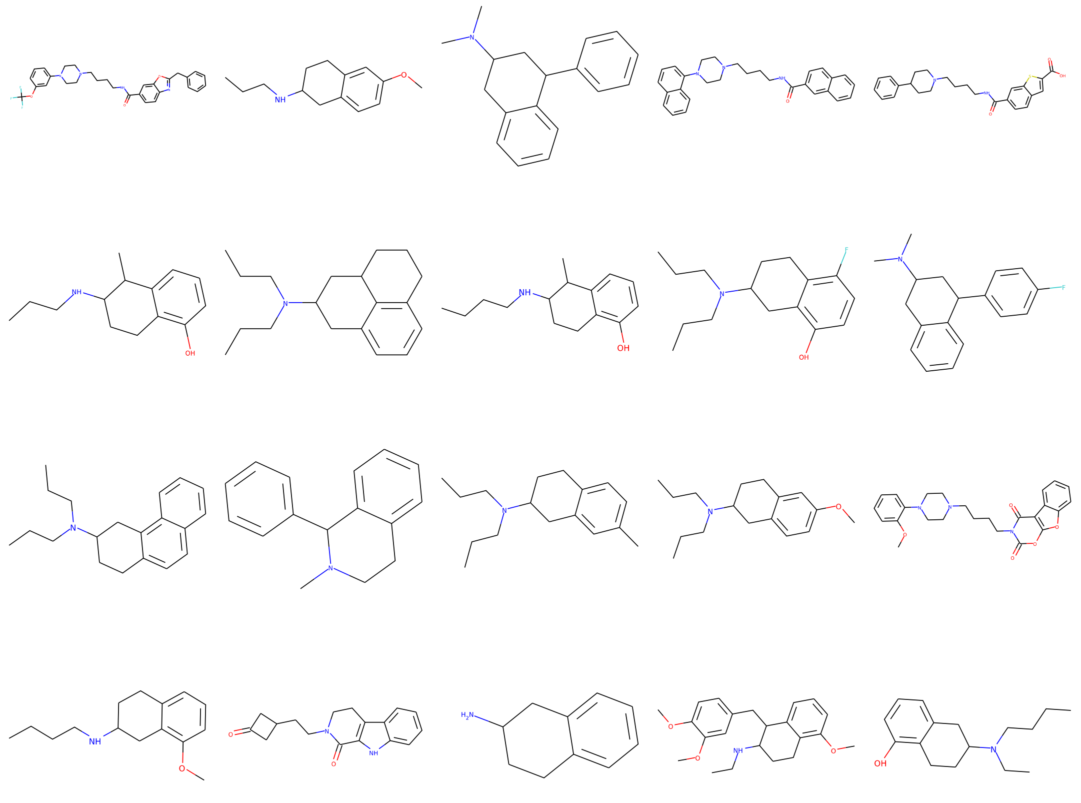

Figure 13 Additional file 2.4 Randomly selected structures generated by the Agent based on the reduced Prior trained to design actives against DRD2.
